# “On the Law of the Sexes;” or the Production of the Sexes at Will

**Published:** 1867-01

**Authors:** Joseph Le Cunte

**Affiliations:** Prof. of Chemistry and Geology in the University of South Carolina, Columbia, S. C.


"*On the Law of the Sexes;*" *or the Production of the Sexes at Will.* By Joseph Le Cunte, M. D., Prof. of Chemistry and Geology in the University of South Carolina, Columbia, S. C.

The following is a very brief extract, condensed from the American Journal of Science and Arts, for July, 1864, and Jan. 1865, of an important memoir of M. Thuny, of Geneva, and of an account of some experiments of M. M. Coste and Gerlee, on the law of the Sexes. The original memoir of M. Thuny was published in the Bibliotheque Universelle in 1863, but, as we have seen no notice of it in the agricultural or physiological journals of the South, we think the intelligent public, as well as the medical profession, will be interested in this abstract.

M. Thuny was first led to his conclusion by the following well known facts:

1. The fundamental or morphological identity of the sexes. From this he concludes that the difference of sexes is due to slight differences in the process of development of the ovum in its earliest stages.

2. That in plants, (those which are uninsexual,) the character of the sex may be controlled by the management of external agents.

3. That, according to Huber, ova of the Bees, if fecundated early, produce workers, (females,) whilst, if fecundation be retarded until the 22d day, all the eggs deposited produced males.

For these reasons M. Thuny concludes that the sex is determined previous to fecundation, or rather by the maturity of the ovum at the moment of fecundation.

It is well known to physiologists that there is a development, and therefore a history to the ovum previous to fecundation. If no fecundation takes place, the development is arrested at a certain stage, and the ovum perishes: but if fecundation occurs there is a new accession to life's force, which suffices to carry it through all stages of embryonic and extra-uterine life.

Now, according to M. Thuny, during the earlier stages of the anti-fecundation history of the ovum, the sex is female; but if the development continues without fecundation it becomes male. By impregnation the sex is fixed forever. If, therefore, impregnation takes place while the ovum is immature, and its sex therefore, female, the embryo will be female; but if fecundation is delayed until a late period, when the sex of the ovum has become male, then the embryo will become male.

It is easy to see the important practical applications of the law. In uniparous mammalia the ovum leaves the ovary at the beginning of each rutting period in a very immature condition, and passes slowly through the fallopian tubes, the uterus, and finally, if unfecundated, is discharged.

Now, during the whole of this slow passage, the ovum is maturing. If, therefore, fecundation takes place early in the period of heat, the sex of the embryo will be female. If later it will be male.

The period of heat, or generative period (as Thuny calls it), here spoken of, must not be confounded with the *season* of heat, or rutting season. All farmers are aware, that during the season of heat there are regular *periods of exacerbation*, which in the case of the cow occur about every two weeks. These are the generative periods spoken of by M. Thuny. They are really menstrual periods, and, if attentively observed, are found to be always attended with slight menstrual discharges. Now, if M. Thuny is right, fecundation at the commencement of the menstrual period will produce females, and later, will produce males. He does not indicate the exact turning point.

Anxious to subject his theory to the list of disinterested experiments, M. Thuny gave minute directions to M. Cornaz, an intelligent Swiss stock-raiser, and son of the President of the Swiss Agricultural Society. These directions were followed in 29 cases, and in every case, without exception the desired sex was produced. First, in order to propagate the breed of a very fine Durham bull, M. Cornaz wished to get heifers, he made 22 experiments and got heifers every time. He then wished to get a few bulls of half breeds to sell to his neighbors; he made seven experiments and got bulls every time.

In the case of multiparous mammalia and birds, the test is much more difficult, and the results contradictory. M. Thuny's observations lead him to think that in the domestic hen, "the last eggs laid are the cocks of the clutch." He accounts for this by supposing that in each generative period several ova commence to operate together, but are separated from the ovary successively, and therefore at the moment of fecundation, (which takes place in the ovaduct), the last separated are the most mature. M. M. Coste and Gebre on the contrary, find that when several ova are fecundated by *one* copulative act, the first laid eggs produce cocks and the last hens. These results are in accordance with certain observations which are as old as Aristotle. This great naturalist observed that pigeons laid but two eggs, one of which produced a male, and the other a female. The celebrated physiologist, Flourens, confounded these results of Aristotle, and in addition proved that the egg first laid produced the male, and the other the female. These observations of Coste and Gerbe, and of Flourens and Aristotle certainly seem to contradict the theory of M. Thuny on hens; but that may be accounted for on his theory, by supposing that during a single generative period, several ova commence to develope successively, and separate successively at the same stage of development, and continue their development in the oviduct previous to fecundation. Being thus regularly arranged in the oviduct in the order of their ages, and therefore of their maturity. If all are fecundated by one copulative act, the most mature, or the males would be laid first. Embryologists must settle the important questions we have started. If definitely settled, then it would seem that experiments on hens were best adapted to test M. Thuny's theory; but until definitely settled, experiments on multiparous animals will avail little. In the meantime the experiments of M. Cornaz on cattle have never been controverted.

Such is a brief extract of the memoir of M. Thuny, and of the experiments of M. M. Coste and Gerbre, intermingled, however, with some explanations of our own, in order to make the whole more intelligible. We would like to see the subject taken up by some of our intelligent stock-raisers. The great importance of the theory, if true, both in a scientific and practical point of view—both to the physiologist and the farmer, can not be doubted. But the history of the theory can only be accomplished by intelligent and very careful observers. The physical signs of the generative period differ in the different species, and in different individuals of the same species, particularly in domestic animals. It is always well marked in wild animals, but in domestic animals it is often obscure. Close and patient observations will, however, overcome all these difficulties.—*Nashville Journ. Med. and Surg.*

